# Pediatric cochlear implantation: The impact of frequency-to-place mismatch after a three-year follow-up

**DOI:** 10.1371/journal.pone.0333088

**Published:** 2025-10-31

**Authors:** Nezar Hamed, Asma Alahmadi, Isra Aljazeeri, Yassin Abdelsamad, Hanaa Alotaibi, Abdulrahman Hagr, Medhat Yousef

**Affiliations:** 1 Department of Otolaryngology-Head and Neck Surgery, College of Medicine, King Saud University, Riyadh, Saudi Arabia; 2 King Abdullah Ear Specialist Center (KAESC), King Saud University Medical City, King Saud University, Riyadh, Saudi Arabia; 3 Department of Otolaryngology, Aljaber Ophthalmology and Otolaryngology Specialized Hospital, Ministry of Health, Ahsa, Saudi Arabia; 4 Research Department, MED-EL GmbH, Riyadh, Saudi Arabia; 5 Audiology Unit, ENT Department, Menoufia University, Menoufia, Egypt; Hannover Medical School: Medizinische Hochschule Hannover, GERMANY

## Abstract

**Introduction:**

Frequency-to-place mismatch in cochlear implants (CIs) may influence auditory and speech outcomes, yet its impact on pediatric patients remains underexplored. This study aims to assess the impact of frequency-to-place mismatch on hearing and speech outcomes.

**Materials and methods:**

In this retrospective study, the angular insertion depth and center frequency of each electrode contact were derived. The difference between the tonotopic and default frequency was used to estimate the mismatch. The impact of this mismatch on auditory and speech outcomes was assessed in pediatric CI users.

**Results:**

This study included 89 implanted ears of young children. The analysis revealed a significant difference between default and postoperative electrode frequencies, with greater mismatches observed in shorter electrode arrays. A weak but significant correlation was observed between mismatch and sound field-aided thresholds (SF-AT), while no clear trends were found in other outcome measures. Patients with lower mismatches tended to perform better, while those with mismatches exceeding 7 semitones showed slightly lower—though non-significant—speech performance. However, a significant difference was observed in SIR, favoring the lower mismatch group.

**Conclusion:**

Children with <7 semitones of fequency-to-place mismatch showed better, though non-significant, outcomes across multiple measures—including SDS, CAP, and SF-AT—and a significant difference in SIR. Despite no significant linear correlations overall, these findings suggest that specific mismatch levels may still influence outcomes after three years of CI use. Future studies should investigate whether tonotopic-based mapping improves speech perception and overall auditory performance in young CI users.

## Introduction

The cochlear implant (CI) is a significant innovation in the medical field, providing an opportunity for individuals with severe or profound sensorineural hearing loss (SNHL) to restore hearing, particularly in children with prelingual deafness. This innovative intervention has transformed auditory rehabilitation, empowering individuals with profound hearing impairments to effectively detect and understand sounds in a manner that significantly enhances their communication and speech performance [1]. Early implantation is crucial, as the developing auditory system relies on timely and consistent auditory input to establish neural pathways essential for speech perception and language acquisition [2]. Although the auditory cortex is highly plastic during early childhood, evidence suggests that this plasticity may not fully compensate for atypical auditory input such as frequency-to-place mismatch, especially during critical periods for language acquisition. Studies have shown that mismatched input during these periods can lead to suboptimal auditory cortical organization and delayed speech development [3,4]. Recent findings also show that mapping strategies designed to reduce such mismatches can enhance speech perception in quiet and noisy environments [5–7].

CI programming is a crucial juncture in the rehabilitation process following surgery. As a standard practice, the CI audio processor is programmed based on the default settings, wherein each electrode delivers specific frequency bands to the adjacent neurons in a logarithmic manner [8]. This default setting mirrors the natural tonotopy of the cochlea, with higher frequencies mapped to the basal electrodes and lower frequencies to the apical ones, irrespective of the actual position of each electrode contact relative to the basilar membrane [9]. However, this standard programming approach does not consider the unique anatomical variations in each patient’s cochlea, which can lead to a frequency-to-place mismatch, where the assigned frequencies do not align with the cochlear regions’ natural frequency sensitivity. Previous studies suggest that frequency-to-place mismatch can negatively impact CI outcomes, encompassing speech perception, language development, and overall auditory performance [10,11]. However, most of these studies have focused on adult populations, where auditory pathways are less plastic. The degree to which these findings apply to prelingual children remains uncertain. Additionally, factors such as the patient’s age at the time of CI surgery, consistent use of the device, and hearing age can substantially impact auditory performance [12–16].

Recent advancements have demonstrated the feasibility of calculating the tonotopic frequency (TF) for each electrode by measuring the cochlear parameters utilizing preoperative or postoperative imaging [17–22]. X-ray imaging has been proposed as a reliable, low-cost alternative to CT, offering reduced radiation exposure while maintaining strong concordance with CT-measured angular insertion depth (AID) and electrode TF [22]. Notably, a substantial discrepancy was observed between the TF and the default frequency (DF) [17–22]. Furthermore, studies have explored the role of spiral ganglion (SG) cells in frequency processing, in addition to the organ of Corti (OC). Stakhovskaya et al. observed significant variation in critical bandwidth distance along the SG, in contrast to the OC [23]. Similarly, Dillon et al. reported improved phoneme recognition with the SG frequency-to-place function, indicating a potentially closer tonotopic alignment. These findings present differing perspectives on the relative importance of OC versus SG mapping in CI outcomes [24]. Such considerations are especially critical for pediatric patients, whose auditory systems are still developing and highly sensitive to the quality and consistency of input. Frequency mismatch in early stages may disrupt normal tonotopic development and delay language acquisition. Thus, identifying and minimizing mismatch in this population is clinically important to optimize outcomes during the sensitive neurodevelopmental period.

Despite growing awareness of the frequency-to-place mismatch and its potential impact on CI outcomes, most studies have focused on adult populations. The consequences of frequency-to-place mismatch in prelingually deafened children over follow-up periods of several years remain underexplored. This study aims to analyze the impact of frequency-to-place mismatch on auditory and speech performance in the pediatric CI population.

## Materials and methods

This retrospective study, approved by the Institutional Review Board at the College of Medicine, King Saud University (Ref. No. 22/0312/IRB), adhered to the ethical guidelines outlined in the Declaration of Helsinki. The data were retrospectively collected from medical reports at our tertiary CI center between August 2022 and February 2023. Informed consent was not applicable as the data were analyzed anonymously.

All pediatric patients aged five years or younger who underwent CI surgery between 2010 and 2019, up to three years of follow-up after CI activation, were considered for this study. The inclusion criteria were as follows: pre-lingual patients with bilateral profound SNHL, availability of preoperative high-resolution temporal bone computer tomography (TB-CT) scans (0.625 mm slice thickness, 230 mAs, 140 kV, rotation time 1 second with 0.3 mm reconstruction in the axial and coronal views), and patients who underwent CI implantation with MED-EL systems (Innsbruck, Austria) with a completely inserted electrode arrays confirmed by post-insertion imaging, which conformed to the surgical planning software utilized (OTOPLAN® software; CASCINATION, Bern, Switzerland). The exclusion criteria included conditions that could affect outcomes, such as cognitive impairments, recent revision surgeries, auditory neuropathy, inner ear anomalies, or any acquired conditions like cochlear ossification, cochlear otosclerosis, or temporal bone fractures involving the otic capsule. It is noteworthy that a previous study revealed a significant difference in the mean cochlear duct length (CDL) of the Saudi population, which was notably shorter compared to European and Australian studies [25]. As a result, CI patients implanted with MED-EL electrode arrays during this period were fitted with different electrode array lengths based on preoperative surgical planning to confirm proper covering of the cochlea. Proper coverage was defined as the electrode array length corresponding to CDL to achieve around 1.5 turns or more [26].

Preoperative TB-CT scans were analyzed using planning software (OTOPLAN 3.0.0 (V4) software; CASCINATION/ MED-EL, AG, Bern, Switzerland) to assess cochlear parameters. These included the A-value (cochlear diameter), B-value (cochlear width), and H-value (cochlear height), calculated based on anatomical landmarks provided by the software. CDL was automatically computed [27]. Complete electrode array insertion was confirmed using postoperative X-ray imaging. Angular insertion depth (AID) and corresponding center frequencies (CF) for each electrode were then calculated following the method described by Alahmadi et al. [28].

The default mapping employed for programming in all CI patients following activation was based on the specific characteristics of the electrode array utilized in this study. This default mapping demonstrated a logarithmic distribution along the electrode array, ensuring that sound frequencies were effectively represented across the cochlea [8]. The central frequencies of the audio processors used ranged from 125 Hz to 8,000 Hz, covering a wide frequency spectrum to facilitate comprehensive auditory perception [21]. This default setting was consistently applied across the CI recipients, aligning with the natural tonotopic organization of the cochlea, where low frequencies correspond to the apical region and high frequencies correspond to the basal turn. However, a recent study by Helpard et al. demonstrated that an individualized frequency mapping function, based solely on the cochlea’s angular length, markedly reduces pitch errors compared to the DF, providing a more precise and personalized method for cochlear frequency mapping [29]. Moreover, measuring the basal turn diameter in preoperative imaging may enhance the accuracy of predicting organ of Corti/ spiral ganglion length and insertion depth, facilitating more precise targeting of angles and frequencies in cochlear implant procedures [23]. The rationale behind this frequency allocation is to optimize speech perception and provide patients with a more natural listening experience, allowing for better sound discrimination and improved speech understanding. The current investigation focused on frequency-to-place mismatch, delineated as the discrepancy between the frequency calculated based on the TF of electrode contact and the DF [21]. This metric was quantified in semitones, representing the smallest musical intervals commonly employed in Western tonal music. A semitone indicates the space between two consecutive notes within a 12-tone scale.

Postoperative audiological and speech assessments were conducted routinely as part of standard follow-up protocols. This study focused on outcomes post-implantation to evaluate the effects observed after three years of CI use of electrode mismatch on hearing and speech development. Preoperative audiological and speech evaluations were not detailed in this manuscript, as all patients had profound SNHL and lacked speech development prior to CI. Audiological outcomes were measured using sound field-aided thresholds (SF-AT) across frequencies from 250 Hz to 8000 Hz. Pediatric-specific testing methods were employed, including visual reinforcement audiometry (VRA), conditioned play audiometry (CPA), and conventional audiometry based on age and cooperation. Speech outcomes were assessed using speech detection thresholds (SDT), speech recognition thresholds (SRT), and speech discrimination scores (SDS). Functional auditory performance was measured using the categorical auditory performance (CAP) test, and speech intelligibility was evaluated using the speech intelligibility rating (SIR). CAP and SIR tests were conducted biannually during the first 36 months post-CI, and annually thereafter. All participants were children aged ≤5 years at the time of implantation. Rehabilitation included regular speech therapy and auditory training to enhance sound discrimination and speech comprehension. CI programming adjustments were made regularly based on developmental progress, aiming to optimize auditory and speech outcomes.

### Statistical analysis

Post-operative cochlear implant data were collected and analyzed using R (version 4.2.2). Descriptive statistics were presented as mean and standard deviation for continuous variables, and as counts and percentages for categorical variables. Normality of data distribution was assessed using the Shapiro–Wilk test. Due to violations of normality assumptions, non-parametric tests were applied throughout. Comparisons between default frequency mapping and post-operative anatomy-based tonotopic frequency (TF) estimates were conducted using the Wilcoxon signed-rank test for paired samples. The absolute frequency-to-place mismatch was calculated in semitones for each electrode channel and averaged across the array. To examine associations between mismatch and post-operative outcomes, the Spearman correlation coefficient was used to assess the relationship between the average semitone mismatch and sound field-aided thresholds (SF-AT). Groupwise comparisons were also conducted between CI recipients with <7 semitones and ≥7 semitones of average mismatch, based on literature identifying 7 semitones as a potential clinical threshold [30]. These comparisons used the Wilcoxon rank-sum test to evaluate differences in SF-AT, SRT, SDS, CAP, and SIR between the two groups.

## Results

The study included a total of 89 cochlear-implanted ears, with a nearly equal distribution between the left (50.6%) and right (49.4%) sides. The mean age of patients at the time of audiological and speech evaluation was 5.8 ± 1.3 years. The average age at the time of implantation was 2.2 ± 1.0 years, while the mean hearing age (months of device use) was 39.1 ± 12.2 months. Patients were implanted with different MED-EL electrode arrays, including FORM24 (51.7%), FLEX28 (39.3%), FLEX26 (3.4%), FORM19 (2.2%), and STANDARD (3.4%), reflecting a range of anatomical and surgical considerations.

Cochlear anatomical measurements showed an average A-value (cochlear diameter) of 8.6 ± 0.5 mm, a B-value (cochlear width) of 6.4 ± 0.5 mm, and an H-value (cochlear height) of 3.6 ± 0.3 mm. The mean cochlear duct length (CDL) across patients was 33.6 ± 2.3 mm. The average angular insertion depth (AID) measured postoperatively using radiographic techniques was 515.4 ± 88.0 degrees, indicating a wide range of electrode insertions across the cochlear spiral. Audiological and speech performance assessments revealed a mean sound field-aided threshold (SF-AT) of 31.5 ± 6.9 dB and an average speech recognition threshold (SRT) of 30.3 ± 6.8 dB. The speech discrimination score (SDS) averaged 68.4 ± 16.8%, suggesting moderate speech perception abilities across the cohort. Functional performance outcomes were also documented, with a mean Categorical Auditory Performance (CAP) score of 5.7 ± 1.5 and a Speech Intelligibility Rating (SIR) of 2.9 ± 1.1. These results are stratified by electrode type in Table 1 for further detail.

**Table 1:** Patient characteristics, cochlear, audiological, and speech outcomes by electrode type.

Patients’ characteristics		Form19 n = 2 (2.2%)	Form24 n = 46 (51.7%)	Flex26 n = 3 (3.4%)	Flex28 n = 35 (39.3%)	Standard n = 3 (3.4%)	Total N = 89 (100%)
**Age at implantation, Years**	Mean (SD)	1.0 (0.0)	2.2 (0.9)	1.0 (0.0)	2.4 (1.1)	1.7 (1.2)	2.2 (1.0)
**Months of device use since first fitting**	Mean (SD)	38.0 (3.5)	39.5 (12.9)	29.0 (0.0)	38.3 (10.8)	61.5 (27.6)	39.1 (12.2)
**Ear**	Left	1 (33.3)	17 (48.6)	1 (50.0)	25 (54.3)	1 (33.3)	45 (50.6)
	Right	2 (66.7)	18 (51.4)	1 (50.0)	21 (45.7)	2 (66.7)	44 (49.4)
**Cochlear parameters**							
**A-Value, mm**	Mean (SD)	8.6 (0.5)	8.8 (0.3)	8.44 (0.54)	8.4 (0.5)	8.8 (0.6)	8.6 (0.5)
**H-Value, mm**	Mean (SD)	3.7 (0.4)	3.6 (0.3)	3.45 (0.46)	3.5 (0.3)	4.0 (0.3)	3.6 (0.3)
**B-Value, mm**	Mean (SD)	6.7 (0.3)	6.6 (0.3)	6.30 (0.61)	6.2 (0.5)	7.3 (0.3)	6.4 (0.5)
**CDL, mm**	Mean (SD)	34.3 (1.2)	34.8 (1.4)	32.44 (3)	32.6 (2.3)	37.1 (1.4)	33.6 (2.3)
**Post-operative angular insertion depth, degree**	Mean (SD)	652.9 (44.3)	515.9 (94.6)	638.1 (1.0)	497.8 (76.5)	562.0 (32.2)	515.4 (88.0)
**Audiological and speech outcomes**							
**Sound field-aided thresholds (SF-AT), dB**	Mean (SD)	36.3 (7.6)	29.8 (5.9)	34.0 (1.4)	32.6 (7.7)	29.7 (1.5)	31.5 (6.9)
**Speech recognition threshold (SRT), dB**	Mean (SD)	35.0 (5.0)	29.7 (7.0)	40.0 (7.1)	30.2 (6.7)	26.7 (2.9)	30.3 (6.8)
**Speech discrimination score (SDS), %**	Mean (SD)	72.0 (4.0)	71.4 (15.8)	--	65.3 (17.3)	66.0 (36.8)	68.4 (16.8)
**Categorical auditory performance (CAP)**	Mean (SD)	6.7 (1.2)	6.0 (1.5)	8.0 (0.0)	5.4 (1.5)	4.3 (0.6)	5.7 (1.5)
**Speech intelligibility rating (SIR)**	Mean (SD)	3.3 (0.6)	3.0 (1.2)	4.0 (0.0)	2.8 (1.1)	1.7 (0.6)	2.9 (1.1)

**CDL**: cochlear duct length**, AID:** angular insertion depth, **SF-AT:** sound field-aided thresholds, **SRT:** Speech recognition threshold, **SDS:** Speech discrimination score, **CAP:** Categorical auditory performance, and **SIR:** Speech intelligibility rating.

**Fig 1:**
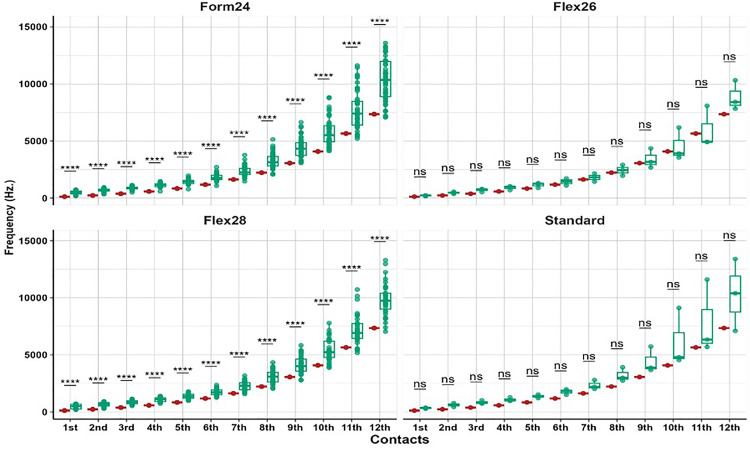
Comparative analysis between the default and post-operative Tonotopic Frequency (TF) for all electrode contacts among different electrode types. (*: 0.05 > p value ≥ 0.01 & ****: p value < 0.001).

**Fig 2:**
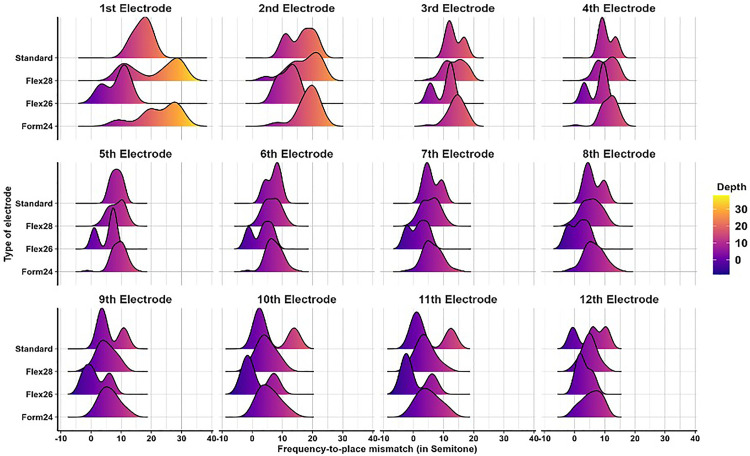
Ridgeline plot for the absolute frequency-to-place mismatch between the default and post-operative Tonotopic Frequency (TF) among different electrode types.

To evaluate the precision of CI programming, frequency-to-place mismatch was quantified by comparing the default frequency assignment of each electrode with its corresponding tonotopic frequency based on the patient’s unique cochlear anatomy. Fig 1 presents a boxplot analysis showing statistically significant differences (p < 0.001) between default and tonotopic frequency assignments across all electrode types. These findings confirm the presence of systematic mismatches between assigned and actual cochlear stimulation sites. Subgroup analysis revealed that shorter electrode arrays were associated with greater mismatches compared to longer electrodes, like the STANDARD array. These mismatches were especially pronounced in the apical and middle electrode regions. No statistically significant difference was observed in the FLEX26 group; however, none of these children were EAS users, and the lack of significance is likely attributable to the very small subgroup size (n = 3), which limits statistical power and increases the risk of a type II (beta) error. A similar limitation applies to the STANDARD electrode group, which also had a small sample size. The distribution and magnitude of mismatch values across the arrays are illustrated in Fig 2, providing a visual representation of mismatch trends.

**Fig 3:**
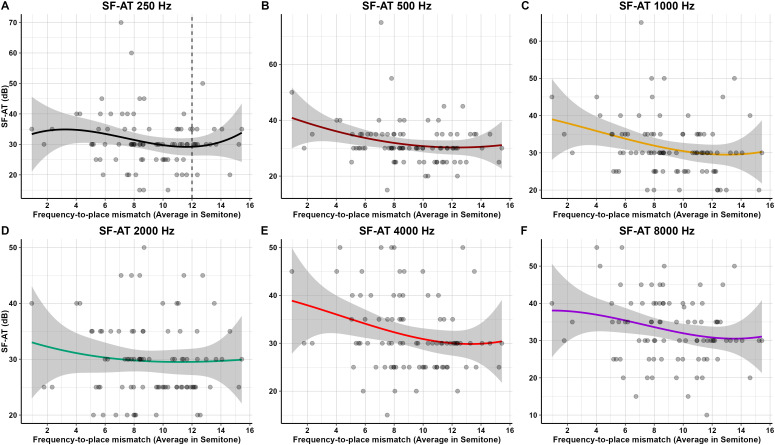
Frequency-specific correlation between mismatch and SF-AT.

Fig 3 illustrates the frequency-specific correlations between frequency-to-place mismatch, defined as the semitonal difference between the default stimulation frequency assigned to an electrode and the tonotopically appropriate frequency based on individual cochlear anatomy, and sound field-aided thresholds (SF-AT) across six audiometric frequencies. At 250 Hz and 500 Hz (Panels A and B), the correlation was minimal and non-significant, with no apparent directional trend. A slight negative association began to emerge at 1000 Hz (Panel C), becoming most apparent at 2000 Hz (Panel D), where the correlation reached statistical significance (r = –0.23, *p* = 0.035)—indicating that the greater mismatch at this frequency was slightly associated with worse aided thresholds. At 4000 Hz and 8000 Hz (Panels E and F), the relationship again diminished, with no significant associations observed. Overall, the data demonstrate a weak and inconsistent relationship between frequency-to-place mismatch and aided thresholds, most evident in the mid-frequency range.

**Fig 4:**
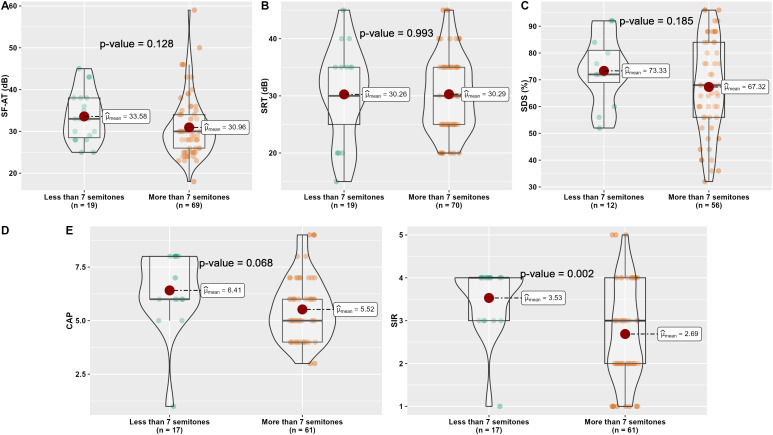
Comparative analyses for the audiological outcomes between CI subjects with less than 7 vs. more than 7 semitonal mismatches. (A) for Sound field-aided thresholds (SF-AT), (B) for Speech recognition threshold (SRT), (C) for Speech discrimination score (SDS), (D) for Categorical auditory performance (CAP), and (E) for Speech intelligibility rating (SIR).

To further examine the potential impact of mismatch magnitude on patient outcomes (Fig 4), subjects were grouped based on whether their average frequency-to-place mismatch was less than or greater than 7 semitones. Groupwise comparisons based on the degree of frequency-to-place mismatch (<7 vs. ≥ 7 semitones) revealed observable trends across multiple auditory and speech outcome measures. Children with mismatch values less than 7 semitones consistently showed better performance. Most notably, the mean Speech Intelligibility Rating (SIR) was significantly higher in the < 7 semitone group compared to those with ≥7 semitones (3.53 vs. 2.69, *p* = 0.002). A similar trend was observed in the Categorical Auditory Performance (CAP) score, with higher mean values in the < 7 semitone group (6.41 vs. 5.52), though this did not reach statistical significance (*p* = 0.068).

Speech Discrimination Scores (SDS) were also higher in patients with less mismatch (73.33% vs. 67.32%), but this difference was not statistically significant (p = 0.185). No meaningful differences were found in sound field-aided thresholds (SF-AT: 33.58 vs. 30.96 dB, p = 0.128) or speech recognition thresholds (SRT: 30.26 vs. 30.29 dB, p = 0.993) between the two groups.

Overall, these results suggest that pediatric CI users with smaller frequency-to-place mismatches (<7 semitones) tend to perform better in speech-related measures, particularly in speech intelligibility, even after more than three years of device use.

## Discussion

The findings of this study shed light on various aspects influencing the postoperative outcomes in pediatric CI recipients, particularly focusing on frequency-to-place mismatch and its impact on auditory and speech outcomes. With a substantial sample size of 89 implanted ears, the study demographics reflect a balanced representation within the pediatric group in terms of gender and age at implantation. Our cohort revealed distinct differences in electrode types, AID, and audiological measures. A notable finding was the significant disparity between the tonotopic frequency (TF), derived from the actual AID from postoperative X-rays, and the default frequencies (DF), consistent with prior findings [6,10,21,22]. Moreover, the TFs consistently demonstrated higher values than the DF across the electrode array.

The results revealed a significant variation in frequency-to-place mismatch across different electrode array types, particularly in the shorter arrays (FORM24) compared to longer ones (STANDARD), and this is similar to what has been reported in previous studies [21]. This variation was more pronounced in the apical and middle electrodes. The STANDARD electrode array did not show a significant difference between DF and TF, likely due to more closely matching the natural tonotopic organization of the cochlea. Furthermore, using longer electrode arrays is expected to enhance cochlear coverage and angular insertion depth (AID), thereby reducing mismatch—particularly in the most apical electrodes. These results align with previous studies indicating that longer electrode arrays with deeper insertion angles enhance outcomes post-cochlear implantation for individuals relying on cochlear implants for hearing [26]. This highlights the importance of electrode array selection in minimizing frequency-to-place mismatch and optimizing auditory outcomes.

Despite the observed mismatches, no significant linear correlation was found between average semitone mismatch and sound field-aided thresholds (SF-AT) in pediatric CI users after up to three years of device use. These findings suggest that frequency-to-place mismatch may have a limited impact on basic hearing thresholds and may not strongly influence auditory performance in the long term. These findings align with a study by Mertens et al. [31], which reported that the impact of frequency-to-place mismatch could diminish after months of CI use. A potential explanation for the non-significant linear relationships observed in our study is that most participants had a hearing age exceeding three years at the time of evaluation, with an average of 3.3 ± 1.1 years. This might supports the role of brain plasticity in compensating the mismatch over time following cochlear implantation. Another possible reason for the generally good outcomes despite the mismatch is the use of longelectrode arrays in our cohort. The average AID in this study was 515.4 ± 88.0 degrees, which is considered adequate for providing broad cochlear coverage. Previous studies have shown that AIDs beyond 500 degrees are often associated with better speech outcomes, as they allow for deeper insertion and closer alignment with the natural tonotopic organization of the cochlea [32,33].

However, the groupwise comparisons based on mismatch magnitude further support the potential clinical relevance of frequency-to-place alignment. Children with <7 semitones of mismatch consistently showed better outcomes across multiple measures, including significantly higher SIR and non-significantly higher SDS, CAP, and SF-AT. While not all differences reached statistical significance, the consistent direction of effect suggests that smaller mismatches may contribute to better speech and auditory outcomes.

This study has certain limitations. Primarily, it is important to note that the audiological data in this study were acquired from a pediatric population, which presents additional challenges compared to adult testing. Children may exhibit variability in attention, cooperation, and comprehension during audiological assessments, which can introduce some level of uncertainty and make the results less reliable. To mitigate these challenges, we employed child-specific testing protocols tailored to engage pediatric patients and worked with experienced pediatric audiologists to ensure accurate and consistent measurements. In addition, it exclusively enrolled patients who had received electrodes compatible with the planning software. Another limitation is the absence of early follow-up data, which reflects the challenges of administering consistent audiological and speech assessments in very young children shortly after implantation, as well as the need to standardize postoperative evaluation intervals to ensure comparability across participants. Finally, in specific electrode subgroups, such as FLEX26 and STANDARD arrays, the small sample size reduced statistical power and increased the risk of a type II (beta) error, which may explain the absence of statistically significant differences despite observed mismatches.

## Conclusion

This study found that children with less than 7 semitones of frequency-to-place mismatch demonstrated significantly better speech intelligibility (SIR) and tended to perform better across SDS, although the difference was not statistically significant. These findings suggest that smaller mismatches may be more favorable for speech outcomes, even after more than three years of device use in pre-lingual pediatric CI users. The observed variability in outcomes may reflect the compensatory role of brain plasticity and the challenges of consistent assessment in this population. Additionally, shorter electrode arrays were associated with greater mismatch, particularly in the apical region, compared to longer arrays—highlighting the importance of proper electrode lenght selection in minimizing mismatch. Future research could explore how different mapping strategies or frequency allocation adjustments might help to further reduce mismatch effects in children.

## Supporting information

S1 File Data. (XLSX)
